# Research Ethics Committees in Africa: Building Capacity

**DOI:** 10.1371/journal.pmed.0040135

**Published:** 2007-03-27

**Authors:** Solomon Benatar

In response to the case study by Kass et al. on research ethics committees (RECs) in Africa [1], the following additional information is provided about capacity building for research ethics in South Africa.

South Africa has two programs funded by the Fogarty International Center: the International Research Ethics Network for Southern Africa (IRENSA; see http://www.irensa.org) based in Cape Town and the South African Research Ethics Training Initiative (SARETI; see http://shsph.up.ac.za/sareti/sareti.htm) that allies the Universities of Pretoria and KwaZulu-Natal. Each of these programs has a different focus and both are making highly valued contributions to capacity building in international research ethics in Southern Africa.

The goal of the IRENSA diploma program is to develop and nourish sustainable multidisciplinary expertise in international research ethics and bioethics in southern Africa. It prepares mid-career health and allied professionals from South Africa and other developing nations in Africa to assume positions of leadership in research ethics in their home institutions. This program is unique on the African continent in focusing exclusively on training mid-career professionals (who cannot take the time or leave to undertake full-time graduate work), in three intensive two-week modules spread throughout one year, with assignments carried out at their home institutions.

In four years IRENSA trained 49 mid-career professionals (17 men, 32 women, 20 white, 29 black) drawn from 20 institutions in South Africa and from 11 institutions in eight other low-income African countries. Sixteen students serve as chairs, deputy chairs, or secretaries of RECs. Students reflect professional training in many disciplines, including science, medicine, nursing, social sciences, law, and pharmacology. Eighteen students hold doctoral degrees and represent a broad spectrum of health organizations. In addition, our annual two-day seminars in research ethics have reached over 400 attendees.

SARETI's goal is to build capacity for ethical review of health research and strengthen Africa's institutional training capacity necessary to achieve and sustain this aim. It offers a multidisciplinary, modular master's degree program with funding for nine trainees over four years, an advanced, non-degree program resulting in a certificate with funding for 16 trainees, and a training program for 40 ethics review committee members. In 2003 SARETI co-hosted, with the HIV/AIDS Vaccines Ethics Group, a two-day training workshop for over 40 members of South African RECs, and in 2004 it offered a three-week Ethics Review Committee Training Program sponsorship to nine South African applicants.

A spin-off of these educational programs has been the formation of a network of Chairs of South African Human Health Research Ethics Committees. This has significantly improved liaison across the country, reduced the potential for shopping around by researchers, and has enhanced the stringency with which protocols are reviewed. A newsletter from Stellenbosch University on research ethics activities in the country draws attention to current debates and events and facilitates networking [2]. The recent stand taken by the chairpersons of RECS in South Africa not to permit studies that do not provide insurance cover for research-related injuries is one example of how improved knowledge and coordination in South African RECs are making such a contribution [3].
